# Dataset from the dynamic shake-table experiments on a full-scale unreinforced clay-brick masonry building with chimneys

**DOI:** 10.1016/j.dib.2023.109813

**Published:** 2023-11-17

**Authors:** Stylianos Kallioras, António A. Correia, Paulo X. Candeias, Alfredo Campos Costa, Francesco Graziotti

**Affiliations:** a University of Pavia, Department of Civil Engineering and Architecture (DICAr), Via Adolfo Ferrata 3, 27100 Pavia, Italy; b European Centre for Training and Research in Earthquake Engineering (EUCENTRE), Via Adolfo Ferrata 1, 27100 Pavia, Italy; c National Laboratory for Civil Engineering (LNEC), Avenida do Brasil 101, 1700-066 Lisbon, Portugal

**Keywords:** Dynamic response, Flexible diaphragm, Non-structural masonry elements, Random vibration test, Sensor measurement, Shake-table test, Structural collapse, Unreinforced masonry

## Abstract

This data paper outlines detailed information on the acquisition and use of sensor measurements from shake-table experiments on a full-scale unreinforced masonry building. The tests were carried out at the shake-table facilities of the National Laboratory for Civil Engineering in Lisbon, Portugal. The building specimen, replicating a typical Dutch single-storey detached house, was made of solid clay bricks and featured a gambrel roof and two chimneys. It was densely instrumented with accelerometers, potentiometers, and LVDTs, recording the response of various structural and non-structural elements. A series of unidirectional dynamic tests of increasing shaking intensity was performed, providing a unique dataset that captures at full scale the in-plane and out-of-plane behaviour of walls and the influence of flexible timber diaphragms on the dynamic global response of an entire building. The dataset is instrumental in improving the accuracy and reliability of simulations focused on the dynamic response, progressive damage, and collapse of unreinforced masonry buildings under seismic actions. The authors made this data available to support the development of analytical and numerical models, advancing research in earthquake engineering and performance-based seismic assessment of unreinforced brick-masonry buildings. The comprehensive dataset, including acceleration and displacement time series, is hosted on the *Built Environment Test Data* platform and is freely accessible without any restrictions through https://www.betestdata.eu/, assisting researchers and engineers in effectively utilising the data for further studies.

Specifications Table

**Table:** 

Subject	Civil and Structural Engineering.
Specific subject area	Earthquake engineering.
Data format	Data in .*txt* and .*mat* files, including raw and processed (filtered) time series.
Type of data	Acceleration and displacement time series. This paper contains tables, graphs, and figures that clarify the data processing assumptions and explain how the dataset from the shake-table tests is organised.
Data collection	A full-scale building specimen was subjected to unidirectional dynamic tests by applying recorded ground motion signals at the foundation via a shaking table. The building was a generic unreinforced masonry structure made of solid clay bricks, embodying construction details of detached houses in the Groningen region of the Netherlands. It had a typical Dutch gambrel roof with tall gables, a flexible timber floor diaphragm, and two clay-brick chimneys of different free-standing heights. The shake-table tests were carried out after a maturation period of 45 days following the construction of the masonry walls. The experiment simulated a series of earthquakes of increasing intensity to assess progressive damage, failure modes, and the ultimate capacity of the structure. The input ground motions were compatible with induced seismicity scenarios for the Groningen gas field of the Netherlands. Random vibration tests were performed between the earthquake simulations to determine the evolution of the dynamic properties of the system at each stage of the experiment. The building specimen was densely instrumented with accelerometers, wire potentiometers, and linear variable displacement transducers that monitored the dynamic response of various structural and non-structural components. The dynamic response characteristics of the building were calculated using the in-house software *LNEC-SPA*, and the acquired dataset was further analysed using *MATLAB*.
Data source location	The shake-table tests were conducted at the National Laboratory for Civil Engineering (LNEC) facilities in Lisbon, Portugal (latitude 38.7581, longitude -9.1424). The project was carried out in collaboration with the research group of Masonry Structures from the University of Pavia and the *EUCENTRE Foundation* in Italy (latitude 45.2024, longitude 9.1340). The recorded data is safely stored in the *EUCENTRE* repository and distributed through the *Built Environment Test Data* platform.
Data accessibility	All recorded data (i.e., acceleration and displacement time series) from the tests are hosted on the *Built Environment Test Data* website (https://www.betestdata.eu/). The platform has been developed, maintained, and hosted by the University of Pavia in collaboration with *EUCENTRE*, with technical support from the *GEM Foundation*. **Repository name**: *Built Environment Test Data*. **Data identification number**: 10.7414/EUC-dzrw13f287. **Direct URL to data**: https://www.betestdata.eu/datasets/26/. **Instructions for accessing these data**: The data is available for free access and can be downloaded without any restrictions.
Related research article	Kallioras S., Correia A.A., Graziotti F., Penna A., Magenes G. Collapse shake-table testing of a clay-URM building with chimneys. Bulletin of Earthquake Engineering 18(3), 1009–1048 (2020). https://doi.org/10.1007/s10518-019-00730-0.

## 1 Value of the Data

• This article presents data from a comprehensive experimental study, including one of the first shake-table tests on a full-scale masonry building with chimneys. This experiment can serve as a benchmark in laboratory testing of structures.
• The paper provides extensive information about the adopted instrumentation plan, test procedure, and data processing methods for researchers who wish to replicate the experiments.
• Various sensors monitored the dynamic response of a complex masonry structure at full scale. This wealth of data can be used to calibrate analytical and numerical models to predict earthquake-induced structural and non-structural damage to buildings.
• Researchers can use the information provided with the data to establish relationships between local and global damage limits for the performance-based seismic assessment of unreinforced clay-brick masonry structures.
• The acceleration and displacement recordings can be further analysed to evaluate the effect of flexible diaphragms on the seismic behaviour of entire building systems.
• The obtained measurements can also be used to study the seismic behaviour of non-structural masonry elements, such as chimneys and gable walls.

## 2 Experimental Design, Materials and Methods

### 2.1 Building specimen

**Fig. 1:**
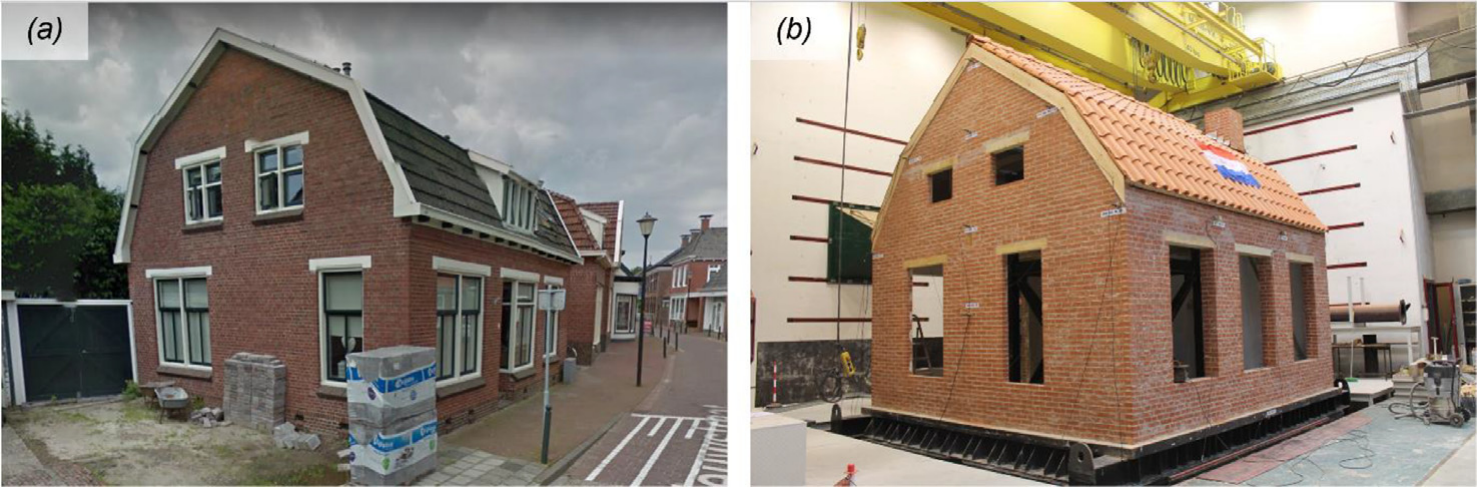
(a) Example of a detached masonry house in Loppersum of the Groningen province in the Netherlands. (b) Full-scale building specimen at the shake-table facilities of LNEC in Lisbon, Portugal.

A series of unidirectional horizontal shake-table tests were performed on a full-scale prototype building (termed *LNEC-BUILD-3*), featuring typical details of pre-1940s detached houses of the Groningen province in the Northern Netherlands (Fig. 1). The specimen consisted of single- and double-wythe unreinforced clay-brick masonry (URM) walls, not detailed for seismic resistance, with large openings in three of the four façades. The floor system consisted of timber beams and planks, providing a flexible diaphragm without any special wall-to-diaphragm connections. The roof structure consisted of timber trusses, purlins, and boards forming a pitched gambrel roof.

The building specimen was subjected to cumulative incremental dynamic excitations, employing two ground motions representative of induced seismicity scenarios for the Groningen gas field. Smooth response spectra and short significant durations characterised the two single-component earthquake accelerograms. The input motions were progressively scaled in amplitude to achieve the desired shaking intensity demands up to the collapse conditions of the building. Random vibration tests were also performed to monitor the evolution of the dynamic properties of the system as a result of cumulative damage.

The geometric characteristics of the prototype building, the construction details, the instrumentation plan, the testing protocol, and the significant observations from the tests are described in detail in the paper of Kallioras et al. (2020) [1]. Therein, the recordings of the sensors have been used to link engineering demand parameters to the attainment of significant performance limit states for assessing the seismic behaviour of clay-brick URM buildings. A series of companion tests were carried out for the mechanical characterisation of the materials employed to construct the specimen. The results of those tests can be found in the *EUCENTRE* research report of Kallioras et al. (2018) [2]. Analysts could use the information provided in the report to generate structural models representative of single-storey detached houses, a building typology widely found in the Netherlands and elsewhere in Northern Europe.

### 2.2 Instrumentation

**Fig. 2:**
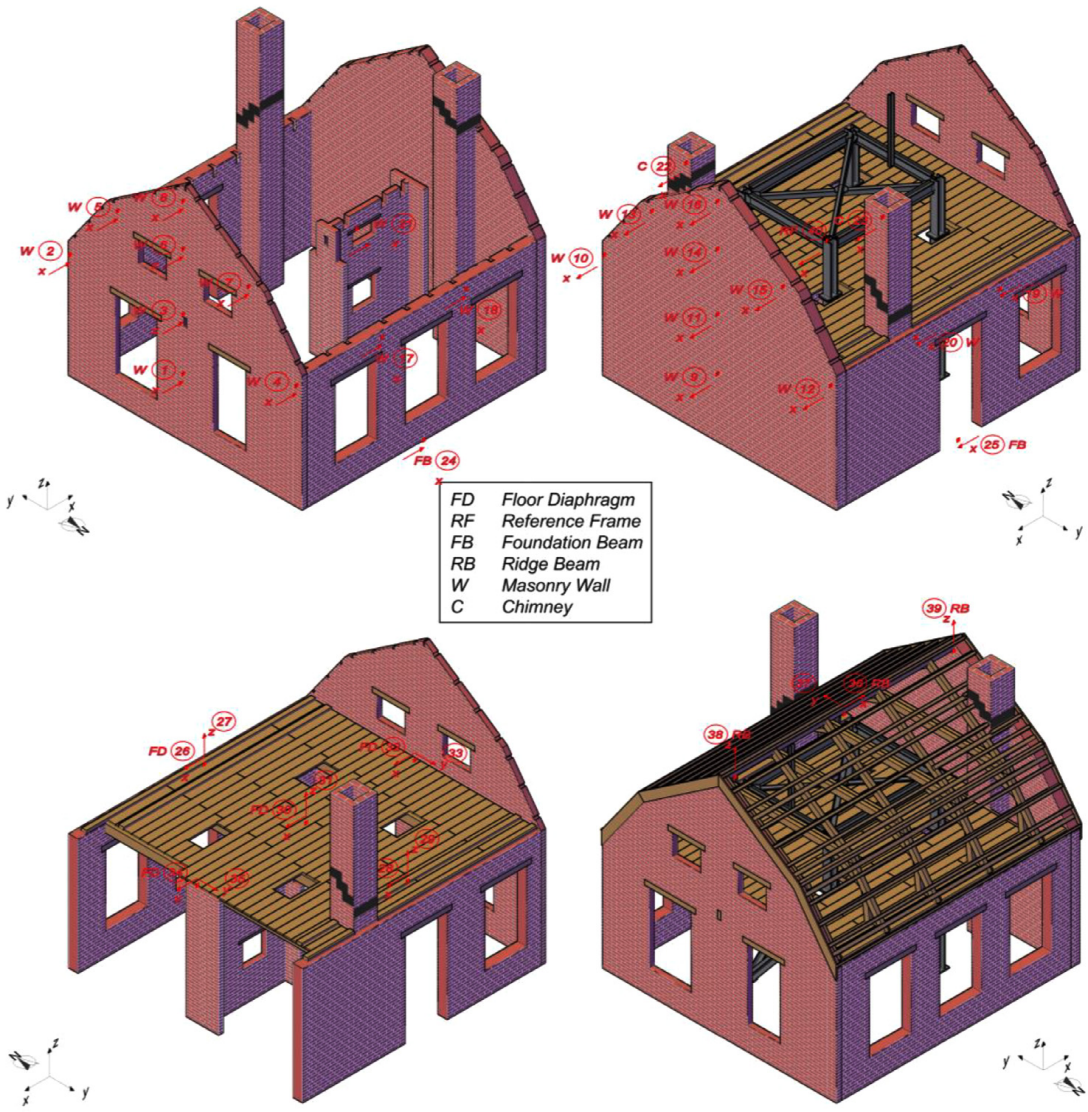
Instrumentation plan featuring unidirectional accelerometers (in red). The labels on the diagram designate the specific structural components where each sensor has been affixed.

**Fig. 3:**
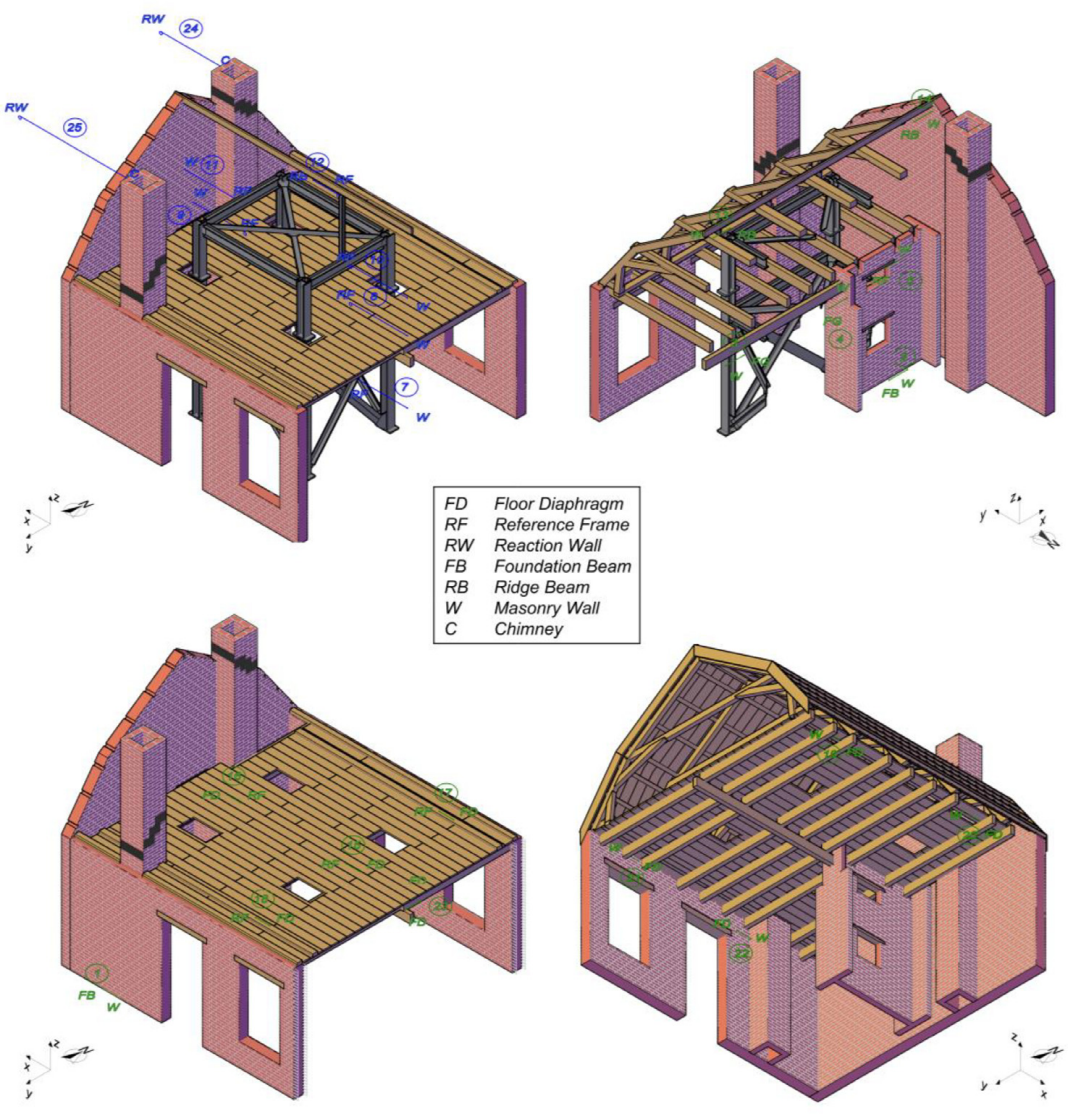
Instrumentation plan featuring wire potentiometers (in blue) and LVDTs (in green). The labels on the diagram designate the specific structural components where each sensor has been affixed.

**Fig. 4:**
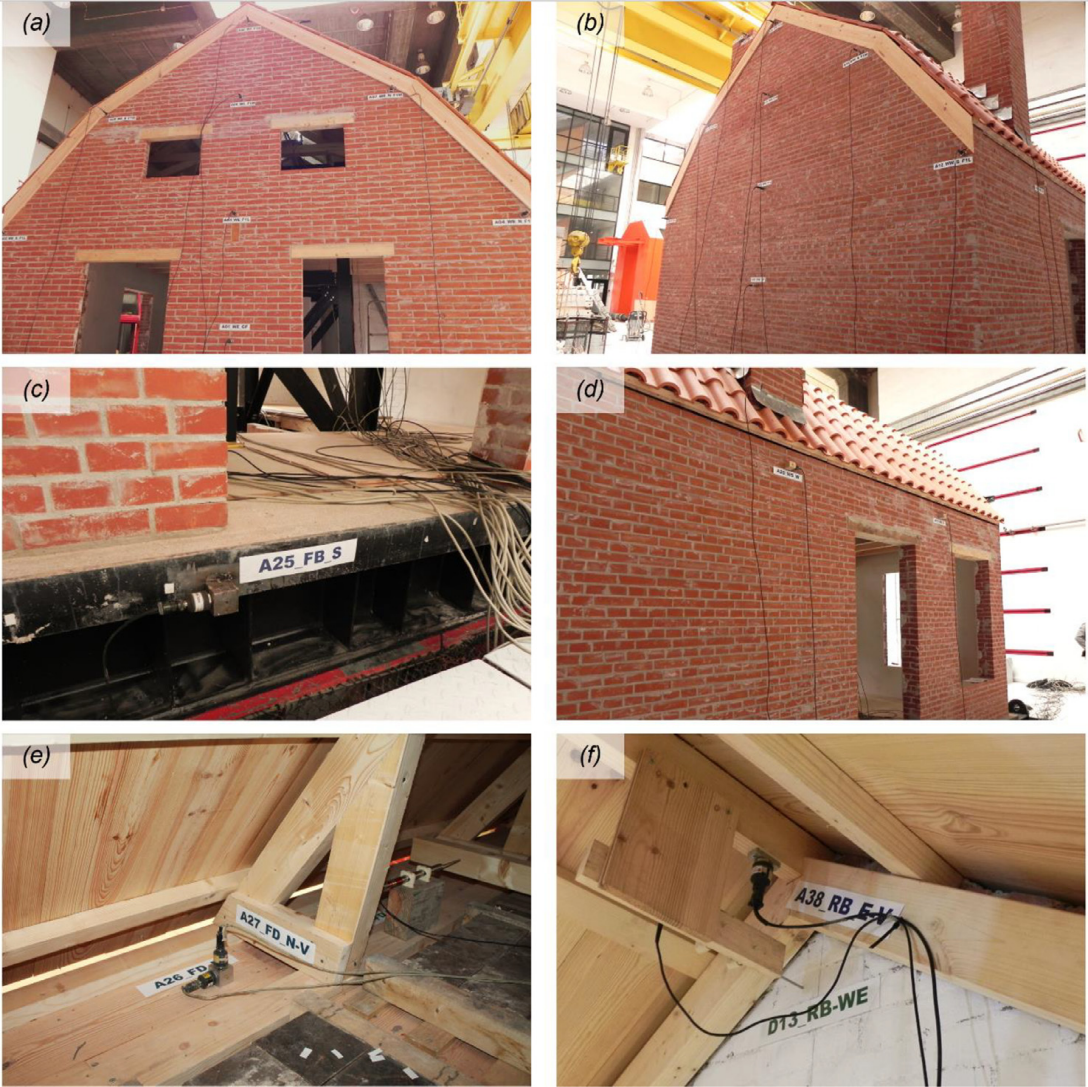
Accelerometers mounted on various structural and non-structural components of the test building: (a) East façade; (b) West façade; (c) foundation beam; (d) South façade; (e) floor diaphragm; (f) roof ridge beam.

**Fig. 5:**
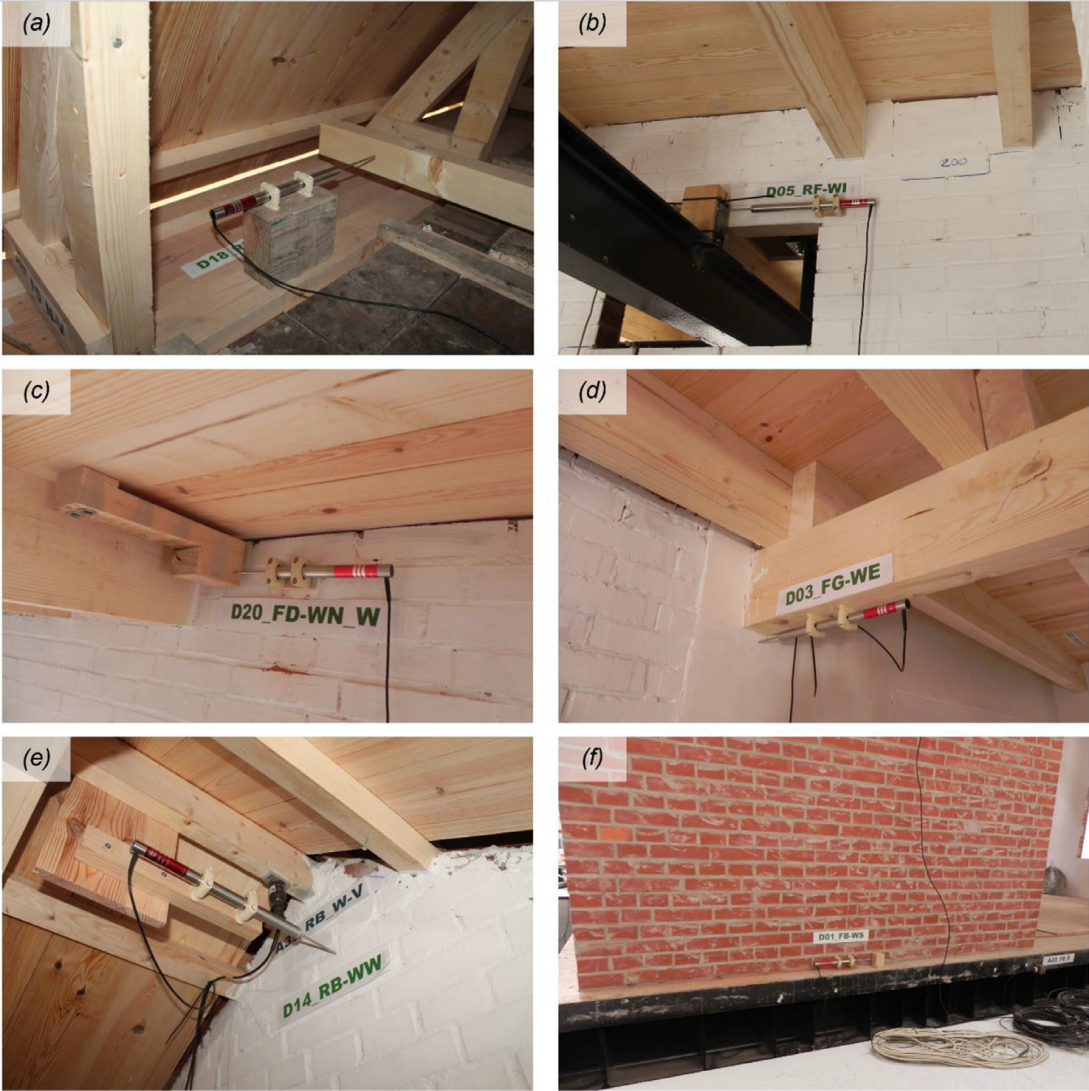
LVDTs monitoring differential displacements between (a) reference frame and floor diaphragm; (b) reference frame and interior wall; (c) South wall and floor diaphragm (i.e., South lower plate); (d) principal floor girder and East wall; (e) West gable wall and roof ridge beam; (f) foundation beam and squat South pier.

**Fig. 6:**
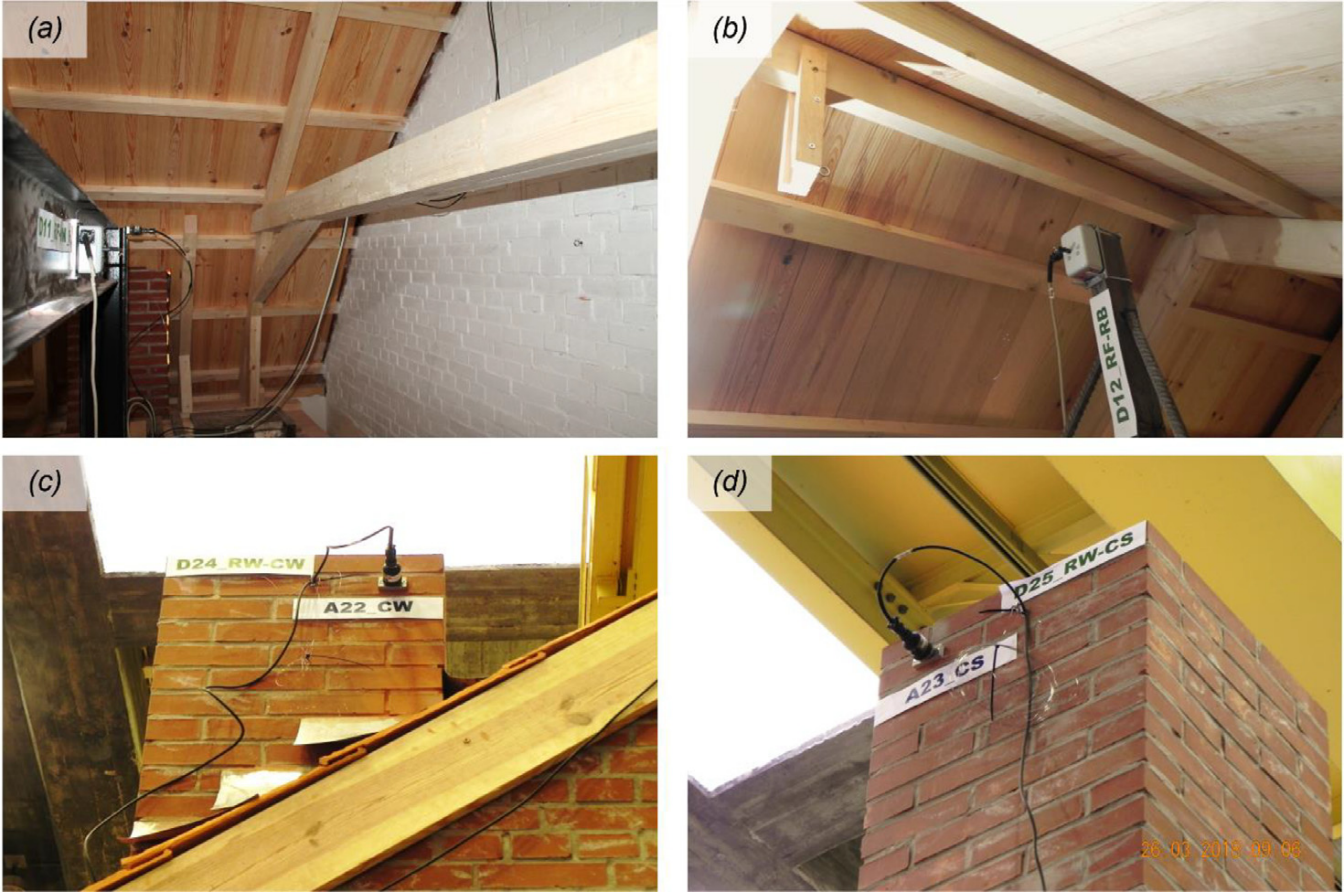
Wire potentiometers recording displacements at (a) mid-height of the West gable wall (w.r.t. the reference frame); (b) roof ridge beam (w.r.t. the reference frame); (c) top of the West chimney (w.r.t. the laboratory reaction wall); (d) top of the South chimney (w.r.t. the laboratory reaction wall).

The instrumentation consisted of 40 accelerometers (A), eight wire potentiometers (WP), and 16 linear variable displacement transducers (LVDTs), which were mounted on the specimen at various locations, as featured in Figs. 2 and 3. Fig. 4, Fig. 5, Fig. 6 offer insightful photographs, spotlighting critical sensors seamlessly integrated into the building. Additional accelerometers and LVDTs were installed on the shake table to record the applied table accelerations and displacements. The earthquake simulation tests were covered by high-definition video cameras installed around and inside the building specimen.

Several instruments were removed while approaching the ultimate limit state of the building to secure them from damage caused by potential falling objects. In most cases, the precautionary measures pertained to accelerometers. Therefore, to calculate the inertia forces, masses at the locations of the affected sensors were allocated accordingly to those instruments that remained mounted on the specimen. In only a few cases, displacement transducers were removed, while in some others, they provided discontinuous readings due to the exceedance of their measuring stroke length (i.e., instrument saturation). Where video recordings were available, the displacement time histories of critical points were retrieved by tracking the motion of the related components using application-specific software (*Tracker*; Open Source Physics, 2020) [3].

For a thorough discussion of the acquired data and the post-processing assumptions, the reader is referred to the section '*Data Acquisition and Processing*' below. A detailed description of the instrument locations, the corresponding measuring quantities, and the structural and non-structural masses associated with each accelerometer is provided in Table 5 of the section ‘*Data Distribution*’.

### 2.3 Test sequence

The building specimen underwent unidirectional dynamic tests that involved applying a series of shake-table motions of increasing intensity. Two single-component, pulse-like earthquake accelerograms (according to the quantitative classification approach by Baker 2007 [4]) were used as input ground motions, representing earthquake scenarios for the Groningen gas field. These two signals were utilised for the shake-table tests of building specimen *EUC-BUILD-2* (refer to Kallioras *et al.*, 2018, research article and dataset) [5,6], which was very similar to *LNEC-BUILD-3*. Initially, the shaking intensity increments were determined based on the experience gained from those tests, while in later stages, the intensity of the input motions was decided by engineering judgment after observing the damage.

The two selected records, SC1 and SC2, were scaled in acceleration amplitude to obtain the desired incremental test sequence, consisting of the 15 earthquake simulations highlighted in grey in Table 1. Between these tests, the building specimen was subjected to 13 low-amplitude random excitations to evaluate the impact of cumulative damage on the evolution of the dynamic properties of the building. Table 1 illustrates the applied test sequence, specifying the ascending test number, test name, input signal, nominal amplitude scale factor of the input accelerogram, and the date and time of execution. Details on the characteristics of the input ground motions and the shake-table performance are documented in Kallioras et al. (2020) [1].

**Table 1:** ummary of the adopted test sequence. Grey lines indicate the earthquake simulation tests (SC1 and SC2); the rest are tests performed for the dynamic identification of the building specimen (RNDM).

Test ID No.	Test name	Input signal	Nominal scale factor	Date	Time
1	CHAR#0	RNDM	–	26 Mar 2018	15:37
2	SC1-50%	SC1	50%	26 Mar 2018	15:48
3	SC1-50%-Rev^†^	SC1	-50%	26 Mar 2018	16:02
4	SC1-100%	SC1	100%	26 Mar 2018	16:06
5	CHAR#1	RNDM	–	26 Mar 2018	16:34
6	SC1-150%	SC1	150%	26 Mar 2018	16:42
7	CHAR#2	RNDM	–	26 Mar 2018	16:57
8	SC2-50%	SC2	50%	26 Mar 2018	17:10
9	SC2-100%	SC2	100%	26 Mar 2018	17:20
10	CHAR#3	RNDM	–	26 Mar 2018	17:36
11	SC2-150%	SC2	150%	26 Mar 2018	17:45
12	CHAR#4	RNDM	–	26 Mar 2018	18:09
13	SC2-200%	SC2	200%	26 Mar 2018	18:15
14	CHAR#5	RNDM	–	26 Mar 2018	18:44
15	CHAR#6	RNDM	–	27 Mar 2018	12:06
16	SC2-100%-Bis*	SC2	100%	27 Mar 2018	12:09
17	SC2-200%-Bis*	SC2	200%	27 Mar 2018	12:52
18	CHAR#7	RNDM	–	27 Mar 2018	13:30
19	SC2-250%	SC2	250%	27 Mar 2018	13:44
20	CHAR#8	RNDM	–	27 Mar 2018	13:58
21	SC2-300%	SC2	300%	27 Mar 2018	16:58
22	CHAR#9	RNDM	–	27 Mar 2018	17:03
23	SC2-350%	SC2	350%	27 Mar 2018	18:58
24	CHAR#10	RNDM	-	27 Mar 2018	19:04
25	SC2-400%	SC2	400%	27 Mar 2018	19:49
26	CHAR#11	RNDM	–	27 Mar 2018	20:04
27	SC2-500%	SC2	500%	27 Mar 2018	20:09
28	CHAR#12	RNDM	–	27 Mar 2018	20:32

^†^The input ground motion was applied with a reversed sign.

*The running earthquake simulation was a repetition of a previous test.

### 2.4 System dynamic identification

A white-noise signal was applied as a low-amplitude random excitation (RNDM) with a dual purpose: (i) to characterise the entire test system (i.e., shake table plus building specimen) during the adaptive tuning process for the target earthquake signals; (ii) to identify the dynamic response properties of the building specimen. In the latter case, the broad-band excitation was used to estimate the variation of the frequency response functions (FRFs) and modal parameters (i.e., modal shapes, natural vibration frequencies and modal damping) of the building with the evolution of structural damage.

The employed input signal was characterised by a nominal peak-to-peak displacement amplitude of 2 mm, a wide-range frequency content, and a sampling rate of 200 Hz, resulting in a total duration of nearly 165 s (precisely, the time series had a length of 32,768 data points). The FRFs were computed based on the input-output relationships for averaged time windows of this signal in a way to reduce the variance of the estimates. To compare the modal analysis results, it was essential always to use input signals of the same type and amplitude.

**Fig. 7:**
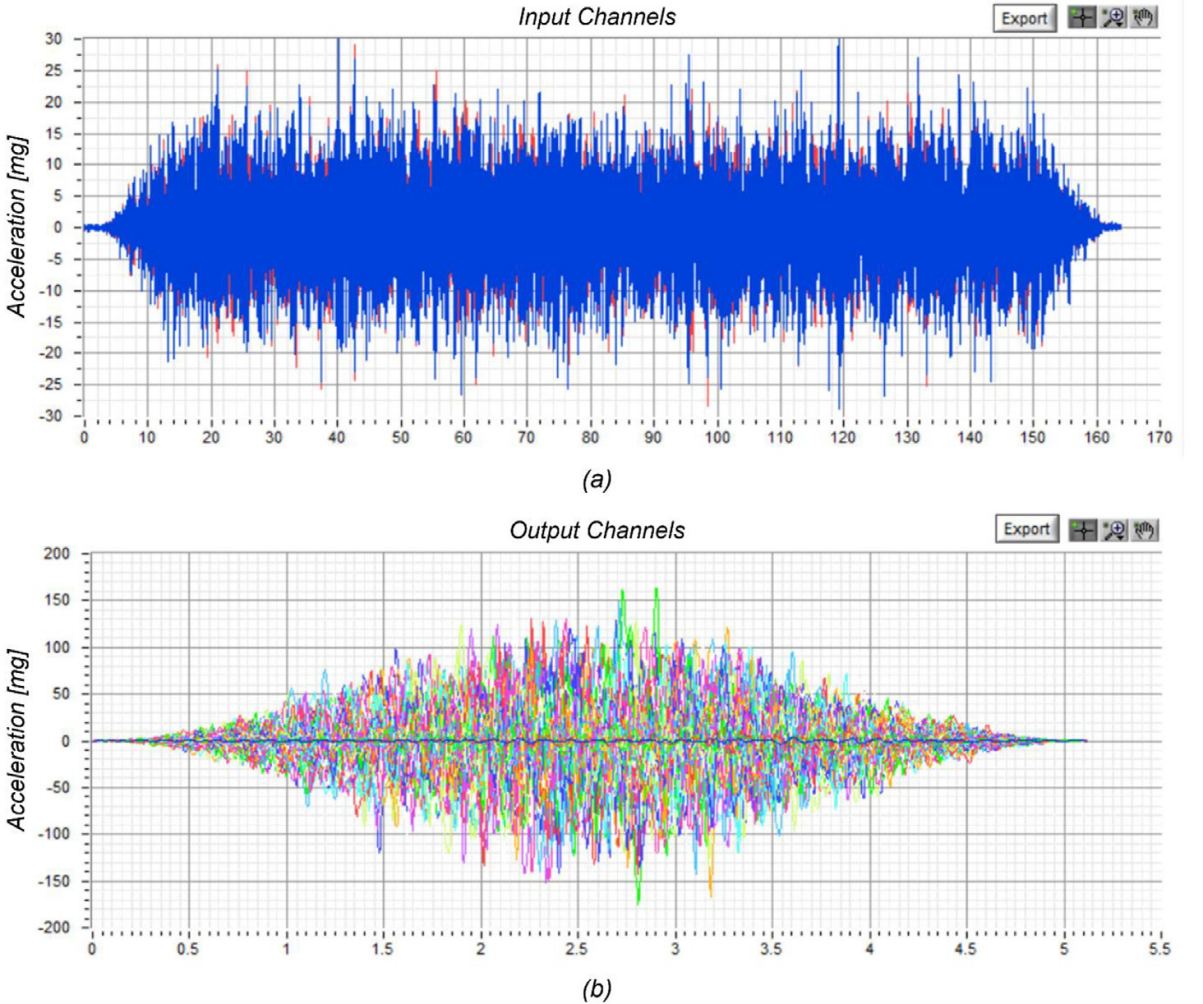
Dynamic identification of the specimen using the *LNEC-SPA* software [7]: (a) input acceleration time series; (b) windowed average output.

**Fig. 8:**
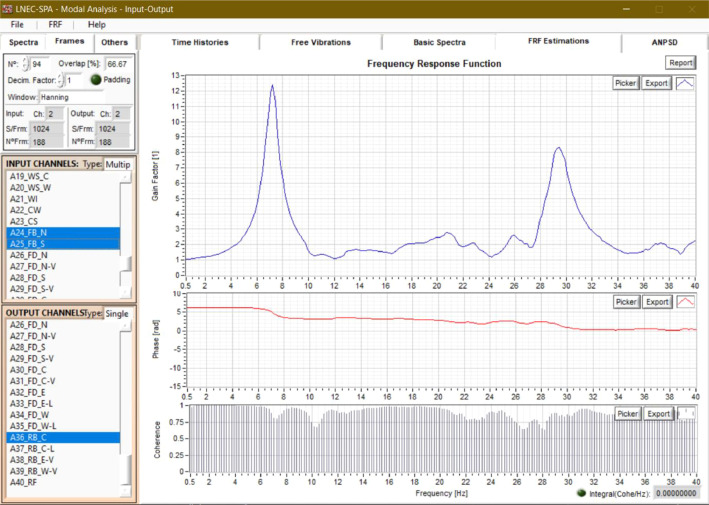
Dynamic identification of the specimen using the *LNEC-SPA* software [7]: frequency response function derived from the accelerations recorded at the roof ridge and the foundation of the building.

The dynamic identification of the building was performed using the acceleration response histories recorded by the accelerometers mounted on the walls, floor and roof of the structure (an example is shown in Fig. 7). The FRFs were determined based on the single-input-to-multi-output relationships between the various acceleration recordings using the *LNEC-SPA* software (Mendes and Campos Costa, 2007) [7]. Fig. 8 shows one of those functions obtained by considering the acceleration at the foundation level as the input signal (average of recordings by A-24 and A-25) and the acceleration at the roof ridge (recorded by A-36) as the output. The complex FRF was computed as the ratio of the cross-spectral density between the input and output signals to the auto-spectral density of the input signal, according to Bendat and Piersol (2010) [8].

The modal frequencies and damping values were estimated using the enhanced frequency-domain decomposition (EFDD) technique, which was proposed by Brincker et al. (2001) [9,10]. The method consists of decomposing the power spectral density matrix into a set of single-degree-of-freedom (SDOF) systems, adjusting the SDOF auto-correlation functions, weighing a set of points in the vicinity of each resonance, and taking them back to the time domain (by inverse Fourier transform). The SDOF auto spectral density functions were identified using the modal assurance criterion (MAC) coefficient, which varied from 0 to 1: unit when the vibration modes had the same configuration and null when orthogonal. The EFDD method allowed for an accurate estimation of the vibration frequencies because it relies on the adjustment to the zero crossings of the auto power spectral density function besides the adjustment to the peaks, which several other factors, such as the frequency resolution, can influence. The modal damping was obtained from the logarithmic decrement of the impulse response function. Nevertheless, it should be noted that it was difficult to compute the damping values accurately. Further details may be found in the *EUCENTRE* research report of Kallioras et al. (2018) [2], available online upon request at https://www.eucentre.it/nam-project.

**Fig. 9:**
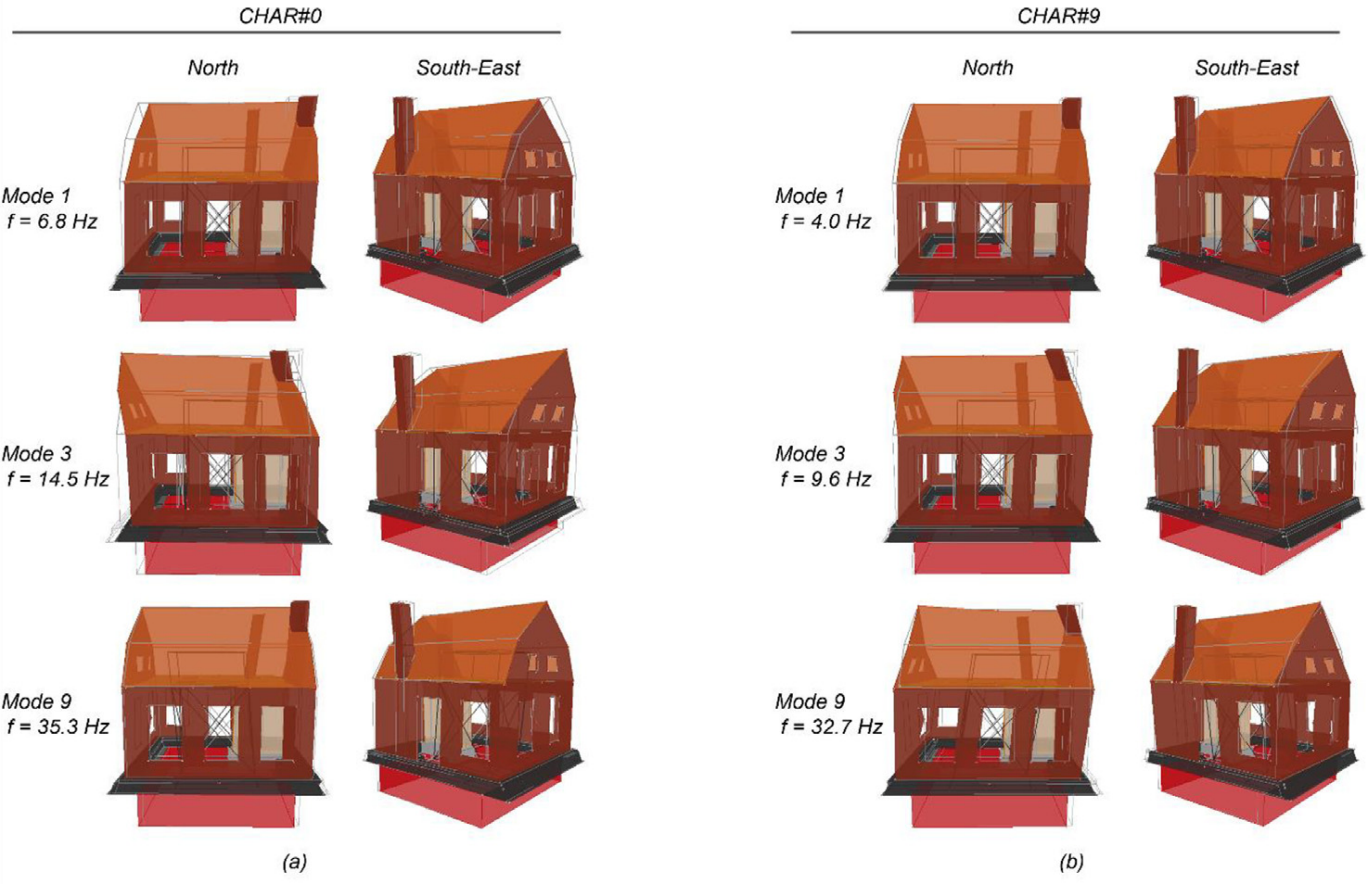
Estimated deformed shapes for the first, third, and ninth modes of the building specimen, (a) at the undamaged state (test CHAR#0) and (b) at a moderate damage state (test CHAR#9).

Table 2 lists the modal properties of the building specimen estimated through the random vibration tests CHAR#0 and CHAR#9, which were conducted prior to any earthquake simulation test and immediately after the earthquake simulation SC2-300%. During these stages, the building was in zero-damage conditions (DS0) and conditions of moderate structural damage (DS3), respectively. In the subsequent testing stages, several accelerometers were removed to protect them from potential damage caused by the building's collapse. The estimates provided in Table 2 indicate a decrease in vibration frequencies and an increase in modal damping ratio as the structural damage severity increased. Fig. 9 displays the estimated deformed shapes for the first, third, and ninth modes of the building specimen, as obtained from the random vibration tests CHAR#0 and CHAR#9.

**Table 2:** Estimated modal parameters for the first nine modes of the building specimen under zero damage (test CHAR#0) and moderate structural damage (test CHAR#9).

	Test CHAR#0 (zero damage state, DS0)			Test CHAR#9 (moderate damage state, DS3)		
Vibration Mode	Frequency	Period	Damping	Frequency	Period	Damping
	[Hz]	[s]	[%]	[Hz]	[s]	[%]
1	6.8	0.147	4.0	4.0	0.251	3.4
2	13.7	0.073	2.5	9.0	0.111	3.4
3	14.5	0.069	2.4	9.6	0.104	3.1
4	17.4	0.058	1.7	14.4	0.070	0.59
5	21.7	0.046	0.41	17.7	0.057	1.7
6	23.4	0.043	1.0	22.1	0.045	0.49
7	27.1	0.037	1.0	23.4	0.043	0.51
8	28.4	0.035	0.22	28.1	0.036	0.70
9	35.3	0.028	0.24	32.7	0.031	0.55

## 3 Data Description

### 3.1 Data acquisition and processing

This section provides information on the sensor measurements from the 15 earthquake simulations and the 13 random vibration tests performed on the building specimen. Some limitations regarding the acquired data are first reported, and then the assumptions in deriving useful response quantities from the recorded acceleration and displacement time series are thoroughly discussed. All datasets have been organised into distributable data files that can be accessed and downloaded freely through the *Built Environment Test Data* platform at the URL https://www.betestdata.eu/. The authors made this information available to encourage the development of analytical and numerical models that simulate the dynamic response of URM buildings with characteristics similar to the *LNEC-BUILD-3* specimen.

Initially, all measurements were acquired at a sampling rate of 5000 Hz; then, decimation was employed to lower the density of the data by a factor of 25. All sensor readings were subjected to low-pass filtering through a Butterworth filter with eight poles limited at 80 Hz. Therefore, the raw measurements are provided with a sampling rate of 200 Hz and usable frequency content up to 80 Hz. The raw dataset, comprising acceleration and displacement time series, was submitted to a further simple post-processing stage, which consisted of filtering out the frequency content above 35 Hz through a low-pass Fourier filter and adding residuals from previous tests to all displacement time series.

#### 3.1.1 Missing instrument recordings

Several instruments were removed while approaching the ultimate damage limit state of the building to secure them from collateral damage due to partial or total collapse. Of the sensors that remained mounted on the specimen, a few accelerometers exhibited intermittent or spurious readings for various reasons, including attachment to collapsed structural or non-structural components (e.g., chimneys), impact with falling objects (e.g., gable bargeboards), or instrument malfunction. Moreover, due to the significant displacement demands on the building during the final shaking runs, several displacement transducers reached their stroke-length capacity, affecting the recorded time series in ranges around the peak displacement responses. Table 3 summarises all sensors that exhibited recording problems or were merely removed to safeguard their integrity.

**Table 3:** List of sensors that were removed or exhibited recording problems during the tests.

Sensor type	Sensor ID No.	Test	Cause of dysfunction
Accelerometer (A)	5, 7, 10, 12, 15, 17, 19, 27, 29, 31, 33, 35, 37, 38, 39	SC2-350%, SC2-400%, SC2-500%; CHAR#10, CHAR#11, CHAR#12	Removal to prevent damage
	22	SC2-400%, SC2-500%; CHAR#11, CHAR#12	Attachment to collapsed component (West chimney)
	13	SC2-350%, SC2-400%, SC2-500%	Impact with a falling object (West gable bargeboard)
	18	Observed during several tests	Sensor intermittent fault
Wire potentiometer (WP)	24	SC2-350%	Instrument saturation
	24, 25	SC2-400%, SC2-500%; CHAR#11, CHAR#12	Removal before imminent instrument saturation
	7	SC2-350%, SC2-400%, SC2-500%; CHAR#10, CHAR#11, CHAR#12	Relocation to another measuring point
	10, 11	SC2-500%	Instrument saturation
Linear variable displacement transducer (LVDT)	19, 20, 21	SC2-350%, SC2-400%, SC2-500%; CHAR#10, CHAR#11, CHAR#12	Removal to prevent damage
	17	SC2-500%	Instrument saturation

#### 3.1.2 Instrument removal or relocation

In total, 15 accelerometers were uninstalled before the test SC2-350% (Table 3), specifically:

i. five accelerometers recording the acceleration response in the *x* (longitudinal) direction at different locations of the East and West building façades (i.e., A-5, A-7, and A-10, A-12, A-15, respectively);
ii. two accelerometers placed on the South and North walls (i.e., A-17 and A-19, respectively), monitoring accelerations in the *x* building direction;
iii. five sensors placed on the floor diaphragm (i.e., A-27, A-29, A-31, A-33, A-35), monitoring accelerations in the *z* (vertical) and *y* (transverse) building directions;
iv. three sensors mounted on the roof ridge beam (i.e., A-37, A-38, A-39), recording accelerations in the *y* and *z* directions.

For the same reason, three linear variable displacement transducers (LVDTs 19, 20, and 21) installed on the South and North walls were uninstalled before the test SC2-350%. These LVDTs monitored the relative displacements of the lower timber plates, which run close and parallel to the South and North edges of the floor diaphragm, with respect to the walls. The amplitude of the corresponding displacement recordings seemed negligible up to that testing phase, and no damage was observed at the connections between floor joists and walls in the following tests. Therefore, the displacements at the top of the North and South walls were reasonably assumed to be equal to those recorded at the floor diaphragm, utilising LVDTs 17 and 18.

The wire potentiometers that measured the displacements at the top of the two chimneys – WPs 24 and 25 – were also dismounted during the last two tests (i.e., SC2-400% and SC2-500%). This was decided because WP-25 had been close to reaching its stroke-length capacity, while WP-24 had already saturated during the test SC2-350% (see section '*Instrument saturation*' below). Without actual measurements, the displacement time histories at the top of the chimneys were retrieved from analysing the video recordings (see Section '*Displacement recordings*').

**Fig. 10:**
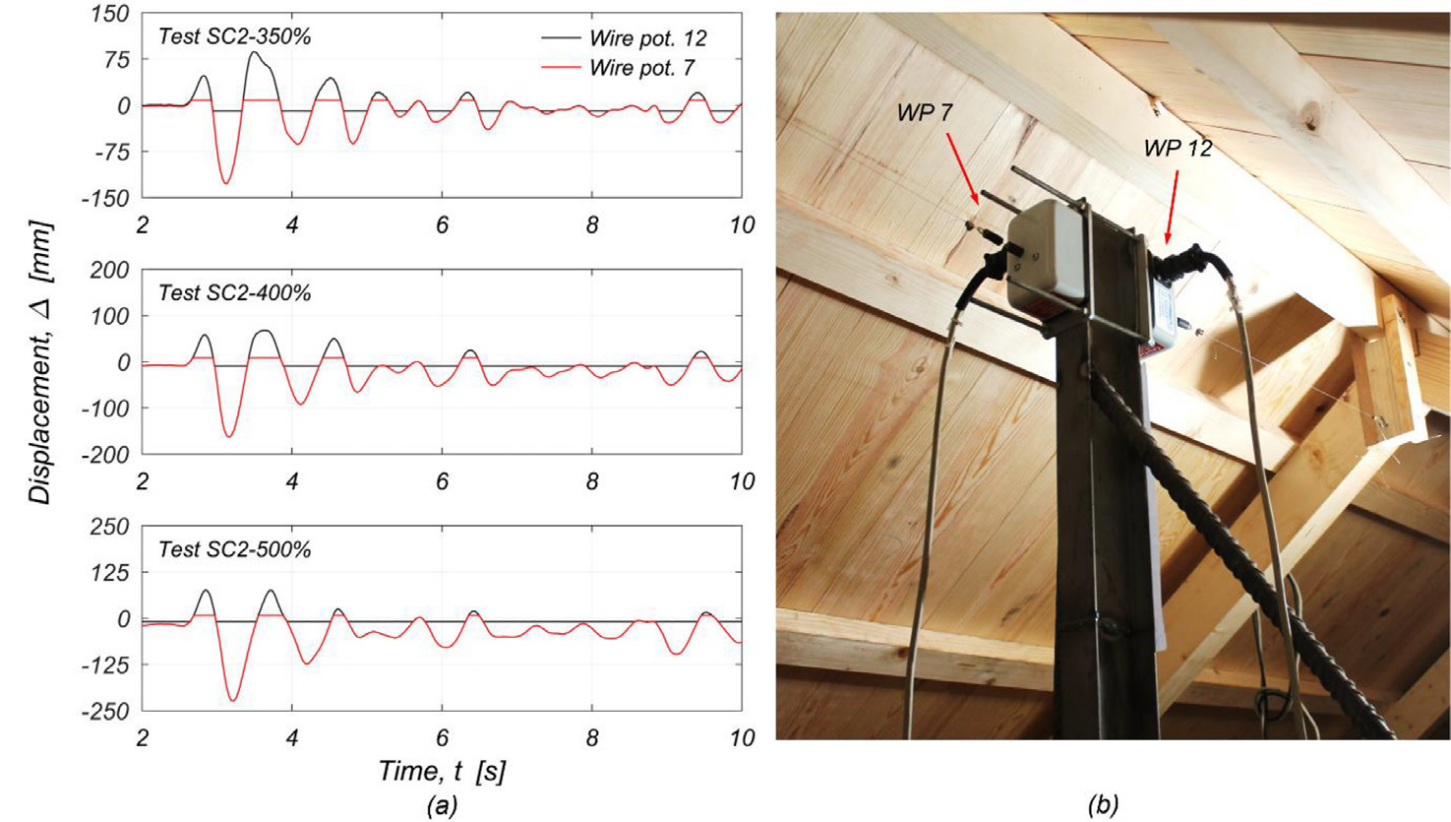
Measuring the displacement of the roof ridge during tests SC2-350%, SC2-400% and SC2-500%: (a) displacement recordings by WP-12 and WP-7 in the time window 2-10 s; (b) location of wire potentiometers WP-12 and WP-7.

Measuring the displacement at the roof ridge was critical. Therefore, during the last three tests (from test SC2-350% onwards), the wire potentiometer WP-12 was adjusted to offer greater stroke length for measuring displacements towards the positive direction (i.e., towards the West). For measuring the displacement in the negative direction, WP-7 was employed (shown in red in Fig. 10a). The instrument initially recorded the out-of-plane deflection of the East middle pier at mid-height of the first storey (see Fig. 3). Consequently, displacement recordings from the latter location are missing from the processed final dataset from test SC2-350% until the end; the corresponding columns were filled with '*not-a-number*' (NaN) elements.

#### 3.1.3 Instrument saturation

**Fig. 11:**
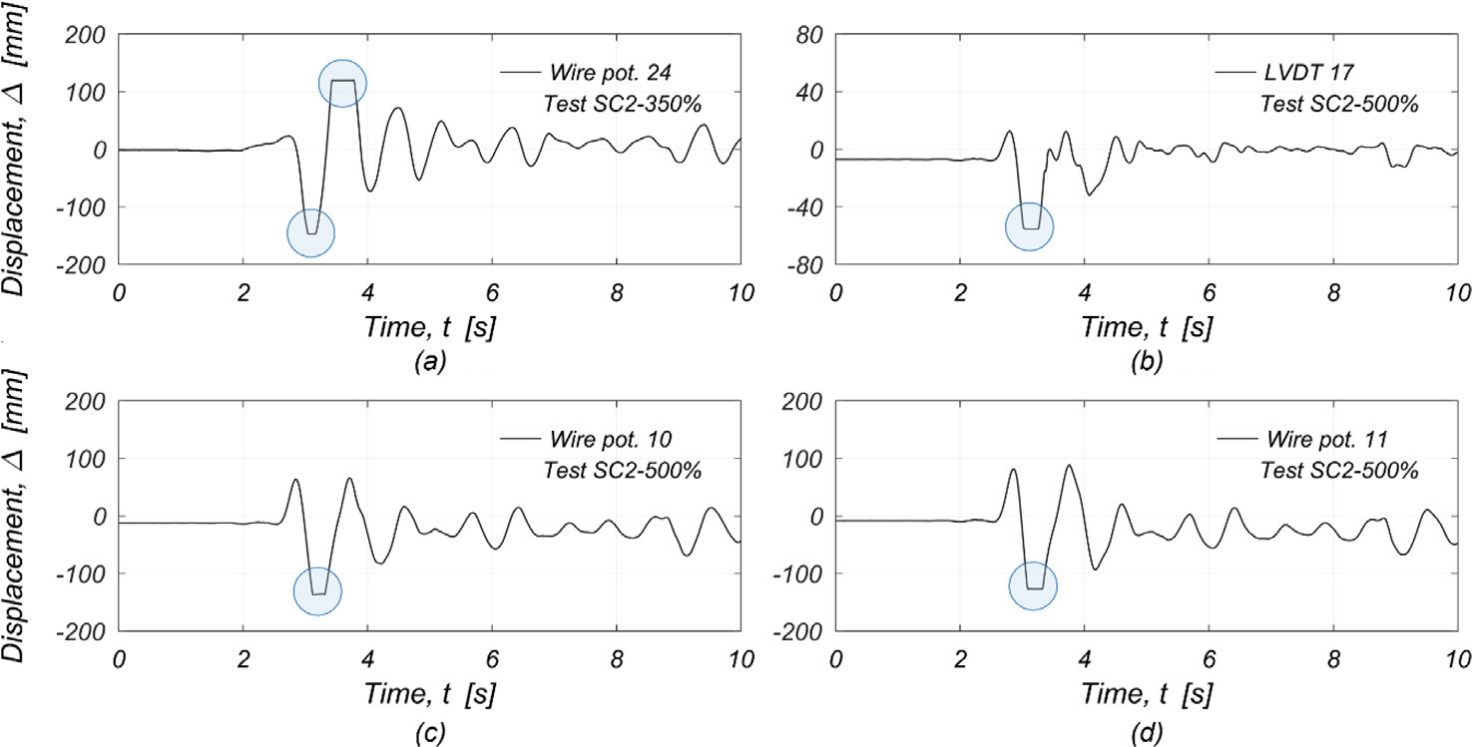
Saturated displacement transducers: (a) WP-24 at the top of the West chimney; (b) LVDT-17 on the North side of the floor diaphragm; (c) WP-10 at mid-height of the East gable wall; (d) WP-11 at mid-height of the West gable wall.
ii. LVDT-17, which measured the relative displacement of the lower plate (found on the North edge of the floor) with respect to the steel reference frame, saturated during the test SC2-500% (Fig. 11b);
iii. WP-10 and WP-11, measuring the out-of-plane displacements at mid-height of the East and West gable walls, respectively, saturated during the SC2-500% shaking run (Fig. 11c and d).
iv. The missing segments of the affected displacement readings were recovered from the analysis of the video recordings of the tests (see Section '*Displacement recordings*').

When the building experienced large displacement demands, several displacement transducers reached their measuring length limits (Table 3). In particular:

i. WP-24, which measured the displacement at the top of the West chimney, reached its stroke-length capacity during testing under SC2-350% (Fig. 11a);

#### 3.1.4 Instrument malfunction

**Fig. 12:**
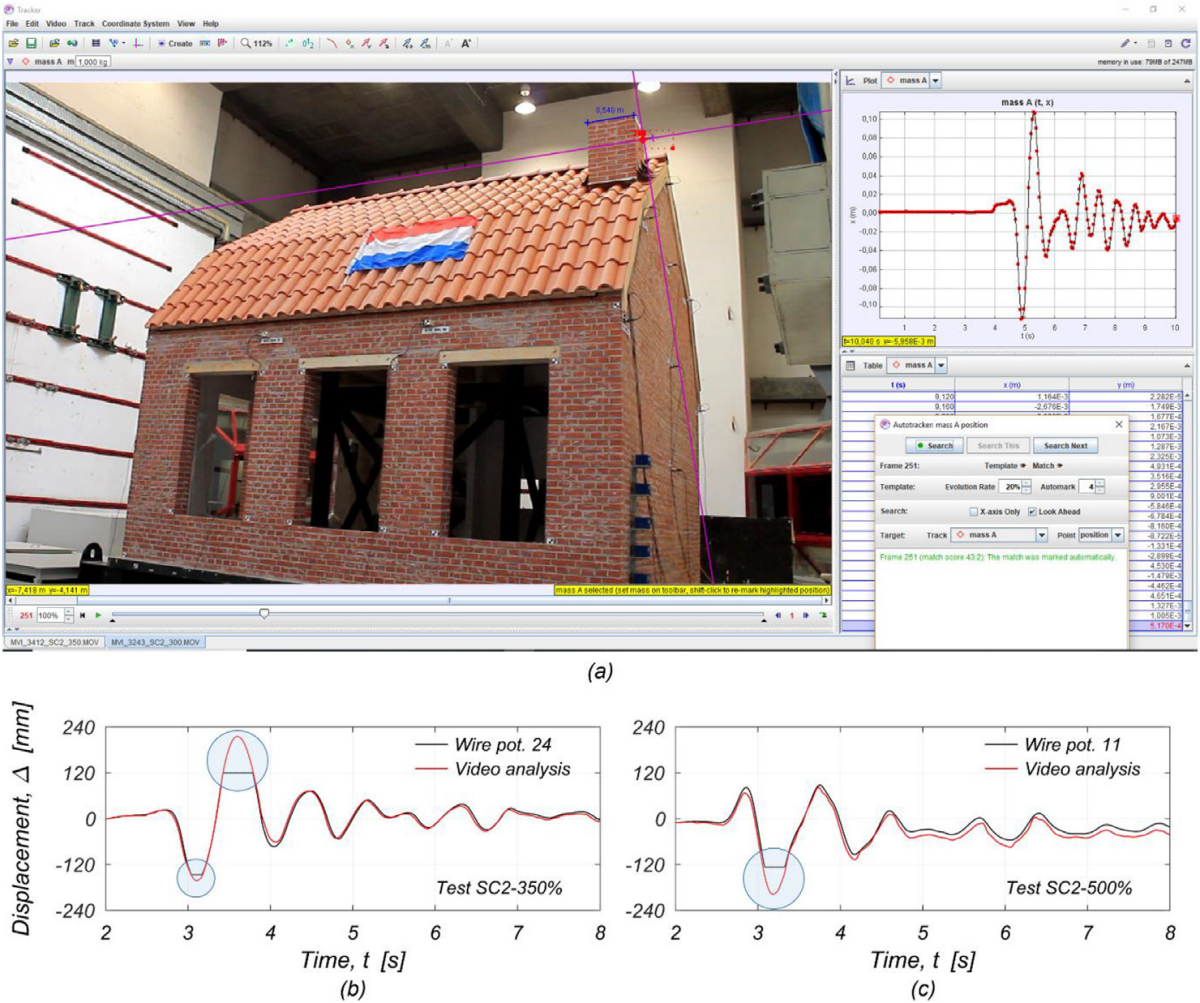
Retrieving missing data using *Tracker* (Open Source Physics, 2020) [3]: (a) example of video analysis of the West chimney for the test SC2-300%; (b) displacement time history of the West chimney during the test SC2-350%; (c) displacement time history at mid-height of the West gable wall during the test SC2-500%.

Accelerometer A-18 was installed on the building specimen throughout all tests. However, the sensor encountered some malfunctions and recorded acceleration time histories with spurious spikes during certain test runs. Therefore, the readings from the instrument were removed from the final processed dataset. To ensure the integrity of other accelerometers near sensor A-18, several of them, including accelerometer A-17 attached to the North masonry wall (as shown in Fig. 12 of the related article [1]), were removed before the last three earthquake simulation tests. As a result, when calculating the developed inertia forces, the accelerations at the top of the North wall were assumed to equal the readings of accelerometer A-17 for testing up to SC2-300%. For tests SC2-350% to SC2-500%, the accelerations were assumed to be equal to those recorded by sensor A-26 at the North edge of the floor diaphragm.

During the test conducted at SC2-350%, issues were also observed with the function of accelerometer A-13. From then on, the instrument recordings included spurious spikes and periods of unstable readings. The sensor was mounted at mid-height of the West gable wall on the Northern side, and the effect was attributed to the impact caused by the collapse of the timber bargeboard attached to the roof purlins on the outer face of the gable wall. For the calculation of the inertia forces, the mass associated with the instrument location was allocated to accelerometer A-14, which was located at the same elevation at the midspan of the gable wall.

#### 3.1.5 Data post-processing

This section discusses the assumptions made in deriving the inertia forces and the critical displacement time series from the raw acceleration and displacement recordings.

##### 3.1.5.1 Acceleration recordings – inertia forces

The building mass was distributed to zones around the accelerometer locations to compute inertia forces. In the absence of several accelerometers during the last test runs, either removed to protect them from damage or exhibiting recording problems due to extensive damage to the specimen, some degrees of freedom were not monitored sufficiently. Thus, structural masses were necessarily redistributed, as detailed below.

i. On the East gable wall, the masses initially associated with accelerometers A-5 and A-7 were allocated to sensor A-6 for the tests SC2-350%, SC2-400% and SC2-500%.
ii. Similarly, on the West gable wall, the masses at the accelerometer locations A-13 (only for the tests displaying malfunction, i.e., from SC2-350% onwards) and A-15 (for all tests) were assigned to A-14.
iii. On the West building façade, instruments A-10 and A-12 (found at the wall edges) were removed before testing at SC2-350%. After that, their tributary masses were associated with accelerometers A-18 and A-20, respectively, found on the North and South return walls.
iv. The wall masses linked to accelerometers A-17 and A-19, installed at the top of the North and South façades, were distributed to the adjacent sensors A-18 and A-20, respectively.

The masses assigned to each accelerometer location (prior to any redistribution mentioned above) are listed in the rightmost column of Table 5 in the section '*Data Distribution*'.

As discussed in the section '*Instrument malfunction*', accelerometer A-18 experienced recording issues during several tests. Consequently, accelerations at that location were considered equal to the readings of accelerometer A-17 for testing stages up to SC2-300%, while they were assumed equal to the accelerations recorded by A-26 at the North edge of the floor diaphragm during tests SC2-350% to SC2-500%. According to the above associations, this assumption affected all inertia forces calculated based on sensor A-18 (meaning instruments A-10 and A-17).

##### 3.1.5.2 Displacement recordings

The displacement time series in the processed datasets include the residuals accumulated during previous earthquake simulation test runs. The residual deformations at the end of every test were computed by averaging the displacement amplitudes within the time window from 1.5 s to 0.5 s before the end of each recording.

Several displacement-recording sensors reached their stroke-length capacity or were removed before imminent saturation (see Table 3). The missing segments of the displacement readings were retrieved from the analysis of the video recordings of the tests, with the use of *Tracker*, a free Java video analysis tool developed by the Open Source Physics Project (available online at https://physlets.org/tracker by OSP, 2020) [3]. An example of such analysis is shown in Fig. 12a for obtaining the displacement time history at the top of the West chimney during the test SC2-300%. The displacement time series obtained from the video recordings sufficiently matched the parts of the records acquired by the laboratory data acquisition system, as evident in Fig. 12b and c.

The Tracker video analysis software enables users to track the movement of an object in a digital video recording, following scale calibration and appropriate coordinate axis definition, much like traditional video analysis. The analysed video recordings are displayed with a sampling frequency of 30 frames per second. As a result, data users must understand that displacement recordings derived from video analysis lack information for vibration frequencies beyond 15 Hz (Nyquist frequency).

#### 3.1.6 Data distribution

The processed datasets from the earthquake simulations are provided in 15 .*txt* files, named after the corresponding shake-table test, as listed in Table 4 (shaded in grey). Each file is a two-dimensional matrix of 105 columns, with each column containing the time series of a measured or derived physical quantity. The lines of the .*txt* files correspond to individual instants of the time series. Similarly, the data from the random-vibration tests are organised in 13 .*txt* files. However, those matrices have just 75 columns because they only provide direct acceleration and displacement measurements from the conventional data acquisition system.

**Table 4:** Shake-table test data: file names.

Test ID No.	Test name	Data file name	Matrix size (rows No. × columns No.)
1	CHAR#0	#1_CHAR#0	35990 × 75
2	SC1-50%	#2_SC1-50%	3990 × 105
3	SC1-50%-Rev	#3_SC1-50%-Rev	3990 × 105
4	SC1-100%	#4_SC1-100%	3990 × 105
5	CHAR#1	#5_CHAR#1	35990 × 75
6	SC1-150%	#6_SC1-150%	3990 × 105
7	CHAR#2	#7_CHAR#2	35990 × 75
8	SC2-50%	#8_SC2-50%	9990 × 105
9	SC2-100%	#9_SC2-100%	9990 × 105
10	CHAR#3	#10_CHAR#3	35990 × 75
11	SC2-150%	#11_SC2-150%	9990 × 105
12	CHAR#4	#12_CHAR#4	35990 × 75
13	SC2-200%	#13_SC2-200%	9990 × 105
14	CHAR#5	#14_CHAR#5	35990 × 75
15	CHAR#6	#15_CHAR#6	35990 × 75
16	SC2-100%-Bis	#16_SC2-100%-Bis	9990 × 105
17	SC2-200%-Bis	#17_SC2-200%-Bis	9990 × 105
18	CHAR#7	#18_CHAR#7	35990 × 75
19	SC2-250%	#19_SC2-250%	9990 × 105
20	CHAR#8	#20_CHAR#8	35990 × 75
21	SC2-300%	#21_SC2-300%	9990 × 105
22	CHAR#9	#22_CHAR#9	35990 × 75
23	SC2-350%	#23_SC2-350%	9990 × 105
24	CHAR#10	#24_CHAR#10	35990 × 75
25	SC2-400%	#25_SC2-400%	9990 × 105
26	CHAR#11	#26_CHAR#11	35990 × 75
27	SC2-500%	#27_SC2-500%	9990 × 105
28	CHAR#12	#28_CHAR#12	35990 × 75

Table 5 describes the content of the first 75 columns of the data matrices for both earthquake simulations and modal identification tests. The columns correspond to quantities directly measured by the sensors. The table lists from left to right: the column number in the data matrix; the sensor identification number; a brief description of the measured quantity and the instrument location; the recorded degree of freedom (DOF); the measurement units (UM); the mass allocated to each accelerometer location. Displacement measurements are expressed in mm units, and accelerations in g units. The data are organised in each file as follows:

i. Column1 provides the time based on a sampling rate of 200 Hz.
ii. Columns 2 to 9 include the displacement and acceleration time histories recorded by two displacement transducers and the six accelerometers permanently mounted on the shake table.
iii. Columns 10 to 33 contain the displacement time histories measured by wire potentiometers and LVDTs. Note that six intermediate instruments indicated in grey in Table 5 were removed or relocated during the last tests (see Table 3), resulting in NaN elements filling up the corresponding time series.
iv. Columns 34 and 35 contain the forces measured by the load cell of the horizontal (longitudinal) and vertical actuators of the shake table, respectively, expressed in kN units.
v. Columns 36 to 75 contain the acceleration time histories recorded by the 40 accelerometers. Note that 16 intermediate sensors indicated in grey in Table 5 were either removed or attached to elements that collapsed in later stages of the testing; consequently, the corresponding columns were filled out with NaN elements (see Table 3).

**Table 5:** Accelerometer and displacement transducer recordings: matrix columns 1 to 75. Letters indicate the measuring instrument: A, accelerometer; WP, wire potentiometer; LVDT, linear variable displacement transducer.

Col. No.	Sensor ID	Measured quantity | sensor orientation and exact location	Rec. DOF	UM	Associated mass (in *x* dir.) [kg]
1	–	Time	–	[s]	–
2	–	Shake-table displacement | *measured in the longitudinal bldg. dir.*	*x*	[mm]	–
3	–	Shake-table displ. | *measured in the transverse bldg. dir.*	*y*	[mm]	–
4	–	Shake-table acceleration | *measured in the longitudinal bldg. dir. (sensor mounted on the South edge of the table)*	*x*	[g]	–
5	–	Shake-table accel. | *measured in the vertical bldg. dir. (sensor mounted at the centre of the table)*	*z*	[g]	–
6	–	Shake-table accel. | *measured in the transverse bldg. dir. (sensor mounted at the centre of the table)*	*y*	[g]	–
7	–	Shake-table accel. | *measured in the longitudinal bldg. dir. (sensor mounted at the North*–*East corner of the table)*	*x*	[g]	–
8	–	Shake-table accel. | *measured in the vertical bldg. dir. (sensor mounted at the North*–*East corner of the table)*	*z*	[g]	–
9	–	Shake-table accel. | *measured in the vertical bldg. dir. (sensor mounted at the South*–*West corner of the table)*	*z*	[g]	–
10	LVDT-1	South squat pier slide | *measured w.r.t. the foundation (sensor mounted at the base of the wall)*	*x*	[mm]	–
11	LVDT-2	Interior wall slide | *measured w.r.t. the foundation (sensor mounted at the base of the wall)*	*x*	[mm]	–
12	LVDT-3	Floor girder slide | *measured w.r.t. the East wall (sensor mounted at the East end of the girder)*	*x*	[mm]	–
13	LVDT-4	Floor girder slide | *measured w.r.t. the interior wall (sensor mounted at the West end of the girder)*	*x*	[mm]	–
14	LVDT-5	Interior wall in-plane displ. | *measured w.r.t. the reference frame (sensor mounted at the floor level of the wall)*	*x*	[mm]	–
15	WP-8	East wall out-of-plane displ. | *measured w.r.t. the reference frame (sensor mounted at the floor level, at midspan of the wall)*	*x*	[mm]	–
16	WP-9	West wall out-of-plane displ. | *measured w.r.t. the reference frame (sensor mounted at the floor level, at midspan of the wall)*	*x*	[mm]	–
17	WP-10	East gable wall out-of-plane displ. | *w.r.t. the reference frame (sensor mounted at mid-height of the gable, at midspan)***For test SC2-500**%**, see column No. 76**	*x*	[mm]	–
18	WP-11	West gable wall out-of-plane displ. | *w.r.t. the reference frame (sensor mounted at mid-height of the gable, at midspan)***For test SC2-500**%**, see column No. 77**	*x*	[mm]	–
19	WP-12	Roof ridge displ. | *measured w.r.t. the reference frame (sensor mounted at the ridge beam)*	*x*	[mm]	–
20	LVDT-13	East gable out-of-plane displ. | *measured w.r.t. the ridge beam (sensor mounted at the ridge level)*	*x*	[mm]	–
21	LVDT-14	West gable out-of-plane displ. | *measured w.r.t. the ridge beam (sensor mounted at the ridge level)*	*x*	[mm]	–
22	WP-25	South chimney displ. | *measured w.r.t. the West reaction wall (sensor mounted at the top of the chimney)***For tests SC2-400**% **and SC2-500**%**, see column No. 78**	*x*	[mm]	–
23	LVDT-23	Floor separation | *measured from joist to joist, along the transverse bldg. dir. ‒ parallel to the East façade (sensor mounted at the ends of the two Easternmost floor joists)*	*y*	[mm]	–
24	LVDT-18	Floor-diaphragm displ. | *measured w.r.t. the reference frame (sensor mounted on the South edge of the diaphragm)*	*x*	[mm]	–
25	LVDT-17	Floor-diaphragm displ. | *measured w.r.t. the reference frame (sensor mounted on the North edge of the diaphragm)***For test SC2-500**%**, see column No. 79**	*x*	[mm]	–
26	LVDT-19	North wall in-plane displ. | *measured w.r.t. the North lower plate (sensor mounted at the floor level, at the North-East wall corner)*	*x*	[mm]	–
27	LVDT-20	North wall in-plane displ. | *measured w.r.t. the North lower plate (sensor mounted at the floor level, at the North-West wall corner)*	*x*	[mm]	–
28	LVDT-21	South wall in-plane displ. | *measured w.r.t. the South lower plate (sensor mounted at the floor level, at the South-East wall corner)*	*x*	[mm]	–
29	LVDT-22	South wall in-plane displ. | *measured w.r.t. the South lower plate (sensor mounted at the floor level, at the South-West wall corner)*	*x*	[mm]	–
30	WP-7	East wall out-of-plane displ. | *measured w.r.t. the reference frame (sensor mounted at mid-height of the first storey, at the wall midspan)*	*x*	[mm]	–
31	WP-24	West chimney displ. | *measured w.r.t. the West reaction wall (sensor mounted at the top of the chimney)***For tests SC2-350**% **and SC2-400**%**, see column No. 80**	*x*	[mm]	–
32	LVDT-15	Floor-diaphragm displ. | *measured w.r.t. the reference frame (sensor mounted on the East edge of the diaphragm, at midspan)*	*x*	[mm]	–
33	LVDT-16	Floor-diaphragm displ. | *measured w.r.t. the reference frame (sensor mounted on the West edge of the diaphragm, at midspan)*	*x*	[mm]	–
34	–	Horizontal shake-table force | *measured by the load cell of the horizontal (longitudinal) shake-table actuators*	*x*	[kN]	–
35	–	Vertical shake-table force | *measured by the load cell of the vertical shake-table actuators*	*z*	[kN]	–
36	A-1	East wall accel. | *measured in the longitudinal bldg. dir. (sensor mounted at mid-height of the first storey, at the wall midspan)*	*x*	[g]	404.6
37	A-2	East wall accel. | *measured in the longitudinal bldg. dir. (sensor mounted at the floor level, at the South-East wall corner)*	*x*	[g]	621.1
38	A-3	East wall accel. | *measured in the longitudinal bldg. dir. (sensor mounted at the floor level, at the wall midspan)*	*x*	[g]	395.2
39	A-4	East wall accel. | *measured in the longitudinal bldg. dir. (sensor mounted at the floor level, at the North-East wall corner)*	*x*	[g]	807.1
40	A-5	East wall accel. | *measured in the longitudinal bldg. dir. (sensor mounted at mid-height of the gable, towards South)*	*x*	[g]	199.5
41	A-6	East wall accel. | *measured in the longitudinal bldg. dir. (sensor mounted at mid-height of the gable, at the wall midspan)*	*x*	[g]	278.6
42	A-7	East wall accel. | *measured in the longitudinal bldg. dir. (sensor mounted at mid-height of the gable, towards North)*	*x*	[g]	199.5
43	A-8	East wall accel. | *measured in the longitudinal bldg. dir. (sensor mounted at the ridge level)*	*x*	[g]	166.8
44	A-9	West wall accel. | *measured in the longitudinal bldg. dir. (sensor mounted at mid-height of the first storey, at the wall midspan)*	*x*	[g]	1626.4
45	A-10	West wall accel. | *measured in the longitudinal bldg. dir. (sensor mounted at the floor level, at the North-West wall corner)*	*x*	[g]	1598.7
46	A-11	West wall accel. | *measured in the longitudinal bldg. dir. (sensor mounted at the floor level, at the wall midspan)*	*x*	[g]	1132.6
47	A-12	West wall accel. | *measured in the longitudinal bldg. dir. (sensor mounted at the floor level, at the South-West wall corner)*	*x*	[g]	1210.4
48	A-13	West wall accel. | *measured in the longitudinal bldg. dir. (sensor mounted at mid-height of the gable, towards North)*	*x*	[g]	711.1
49	A-14	West wall accel. | *measured in the longitudinal bldg. dir. (sensor mounted at mid-height of the gable, at the wall midspan)*	*x*	[g]	673.6
50	A-15	West wall accel. | *measured in the longitudinal bldg. dir. (sensor mounted at mid-height of the gable, towards South)*	*x*	[g]	462.0
51	A-16	West wall accel. | *measured in the longitudinal bldg. dir. (sensor mounted at the ridge level)*	*x*	[g]	369.2
52	A-17	North wall accel. | *measured in the longitudinal bldg. dir. (sensor mounted at the floor level, at the Eastward central pier top)*	*x*	[g]	656.2
53	A-18	North wall accel. | *measured in the longitudinal bldg. dir. (sensor mounted at the floor level, at the Westward central pier top)*	*x*	[g]	657.3
54	A-19	South wall accel. | *measured in the longitudinal bldg. dir. (sensor mounted at the floor level, at the top of the central pier)*	*x*	[g]	765.1
55	A-20	South wall accel. | *measured in the longitudinal bldg. dir. (sensor mounted at the floor level, at the top of the squat pier)*	*x*	[g]	1919.3
56	A-21	Interior wall accel. | *measured in the longitudinal bldg. dir. (sensor mounted at the floor level, at the wall midspan)*	*x*	[g]	751.2
57	A-22	West chimney accel. | *measured in the longitudinal bldg. dir. (sensor mounted at the top of the chimney)*	*x*	[g]	266.7
58	A-23	South chimney accel. | *measured in the longitudinal bldg. dir. (sensor mounted at the top of the chimney)*	*x*	[g]	496.8
59	A-24	Foundation beam accel. | *measured in the longitudinal bldg. dir. (sensor mounted on the North edge of the foundation, at midspan)*	*x*	[g]	4105.9
60	A-25	Foundation beam accel. | *measured in the longitudinal bldg. dir. (sensor mounted on the South edge of the foundation, at midspan)*	*x*	[g]	4281.7
61	A-26	Floor-diaphragm accel. | *measured in the longitudinal bldg. dir. (sensor mounted on the North edge of the diaphragm, at midspan)*	*x*	[g]	1380.5
62	A-27	Floor-diaphragm accel. | *measured in the vertical bldg. dir. (sensor mounted on the North edge of the diaphragm, at midspan)*	*z*	[g]	–
63	A-28	Floor-diaphragm accel. | *measured in the longitudinal bldg. dir. (sensor mounted on the South edge of the diaphragm, at midspan)*	*x*	[g]	1380.5
64	A-29	Floor-diaphragm accel. | *measured in the vertical bldg. dir. (sensor mounted on the South edge of the diaphragm, at midspan)*	*z*	[g]	–
65	A-30	Floor-diaphragm accel. | *measured in the longitudinal bldg. dir. (sensor mounted at the centre of the floor diaphragm)*	*x*	[g]	419.0
66	A-31	Floor-diaphragm accel. | *measured in the vertical bldg. dir. (sensor mounted at the centre of the floor diaphragm)*	*z*	[g]	–
67	A-32	Floor-diaphragm accel. | *measured in the longitudinal bldg. dir. (sensor mounted on the East edge of the diaphragm, at midspan)*	*x*	[g]	419.0
68	A-33	Floor-diaphragm accel. | *measured in the transverse bldg. dir. (sensor mounted on the East edge of the diaphragm, at midspan)*	*y*	[g]	–
69	A-34	Floor-diaphragm accel. | *measured in the longitudinal bldg. dir. (sensor mounted on the West edge of the diaphragm, at midspan)*	*x*	[g]	419.0
70	A-35	Floor-diaphragm accel. | *measured in the transverse bldg. dir. (sensor mounted on the West edge of the diaphragm, at midspan)*	*y*	[g]	–
71	A-36	Roof ridge accel. | *measured in the longitudinal bldg. dir. (sensor mounted at midspan of the ridge beam)*	*x*	[g]	1504.0
72	A-37	Roof ridge accel. | *measured in the transverse bldg. dir. (sensor mounted at midspan of the ridge beam)*	*y*	[g]	–
73	A-38	Roof ridge accel. | *measured in the vertical bldg. dir. (sensor mounted at the East end of the ridge beam)*	*z*	[g]	–
74	A-39	Roof ridge accel. | *measured in the vertical bldg. dir. (sensor mounted at the West end of the ridge beam)*	*z*	[g]	–
75	A-40	Reference frame accel. | *measured in the longitudinal bldg. dir. (sensor mounted at the top South-West corner of the frame)*	*x*	[g]	–

Positive displacements and accelerations indicate motion towards the west side of the building. All acceleration and displacement recordings were filtered using a low-pass Fourier filter set to 35 Hz. The displacement time series obtained from the earthquake simulations include residuals accumulated during previous test runs. In contrast, in the case of random vibrations, the displacement recordings are offset to zero.

The three displacement transducers that became saturated during the test SC2-500% (i.e., WP-10, WP-11, and LVDT-17) are highlighted in red in Table 5, and the corresponding displacement time series have been replaced with NaN elements in the data matrix. Similarly, the displacement recording of potentiometer WP-24, which was affected by the instrument saturation during test SC2-350%, has been replaced with NaN members. The accelerometers that recorded spurious accelerations (i.e., A-13 from test SC2-350% to the end and A-18 during all tests) are also highlighted in red in Table 5.

All displacement time series that have been fully retrieved or complemented with information from the analysis of the video recordings are included in columns 76 to 80 of the data matrix, as described in Table 6. The West chimney collapsed during testing at SC2-400%, so the corresponding acceleration and displacement responses (columns 57 and 80, respectively) are cut at 4.22 s (i.e., time step No. 845).

**Table 6:** Displacement time histories retrieved from the analysis of video recordings for the measuring sensors that exhibited problems: matrix columns 76 to 80.

Col. No.	Sensor ID	Measured quantity | orientation and exact location of dysfunctional sensors	Rec. DOF	UM	Tests
76	WP-10	East gable wall out-of-plane displ. | *measured w.r.t. the shake table (sensor mounted at mid-height of the gable)***For recordings until test SC2-400**%**, see column No. 17**	*x*	[mm]	SC2-500%
77	WP-11	West gable wall out-of-plane displ. | *measured w.r.t. the shake table (sensor mounted at mid-height of the gable)***For recordings until test SC2-400**%**, see column No. 18**	*x*	[mm]	SC2-500%
78	WP-25	South chimney displ. | *measured w.r.t. the laboratory floor (sensor mounted at the top of the chimney)***For recordings until test SC2-350**%**, see column No. 22**	*x*	[mm]	SC2-400%; SC2-500%
79	LVDT-17	Floor-diaphragm displ. | *measured w.r.t. the shake table (sensor mounted at the North edge of the diaphragm)***For recordings until test SC2-400**%**, see column No. 25**	*x*	[mm]	SC2-500%
80	WP-24	West chimney displ. | *measured w.r.t. the laboratory floor (sensor mounted at the top of the chimney)***For recordings until test SC2-300**%**, see column No. 31**	*x*	[mm]	SC2-350%; SC2-400%

Table 7 describes the quantities in columns 81 to 103 of the .*txt* data files from the earthquake simulations. These quantities were not directly measured by the data acquisition system but were derived after post-processing. Inertia forces, such as base shear and gable-roof inertia forces, were computed after the assumptions mentioned in the section '*Acceleration recordings – inertia forces*' and further discussed in Section 7.4 of the related article (Kallioras *et al.*, 2020) [1].

**Table 7:** Derived acceleration, displacement, and force data: matrix columns 81 to 103.

Col. No.	Computed quantity | symbol	DOF	UM	Description/derivation
81	Shake-table accel. | *a*_T_	*x*	[g]	Average of col. 4 and 7
82	Foundation accel. | *a*_g_	*x*	[g]	Average of col. 59 and 60
83	South wall accel. | a_1,S_*(at the floor level of the wall)*	*x*	[g]	Average of col. 54 and 55 until test SC2-300%; Equal to col. 55 from test SC2-350% till the end
84	North wall accel. | a_1,N_*(at the floor level of the wall)*	*x*	[g]	Equal to col. 52 until test SC2-300%; Equal to col. 61 from test SC2-350% till the end
85	Floor-diaphragm accel. | a_1,D_	*x*	[g]	Average of col. 61, 63, 65, 67, 69
86	Roof ridge accel. | a_R_	*x*	[g]	Equal to col. 71
87	West chimney accel. | a_t,C,W_*(at the top of the chimney)*	*x*	[g]	Equal to col. 57
88	South chimney accel | a_t,C,S_*(at the top of the chimney)*	*x*	[g]	Equal to col. 58
89	Base displ. | Δ_g_*(at the shake-table/foundation level)*	*x*	[mm]	Equal to col. 2
90	South floor-diaphragm displ. | Δ_1,S_	*x*	[mm]	Equal to col. 24
91	North floor-diaphragm displ. | Δ_1,N_	*x*	[mm]	Equal to col. 25 until test SC2-400%; Equal to col. 79 for test SC2-500%
92	Average floor-diaphragm displ. | Δ_1,AVG_	*x*	[mm]	Average of col. 24, 25, 32, 33 until test SC2-400%;
93	Roof ridge displ. | Δ_R_	*x*	[mm]	Equal to col. 19
94	West chimney displ. | Δ_t,C,W_*(at the top of the chimney, w.r.t. the base)*	*x*	[mm]	Equal to col. 31 until test SC2-300%; Equal to col. 80 for tests SC2-350%, SC2-400%
95	South chimney displ. | Δ_t,C,S_*(at the top of the chimney, w.r.t. the base)*	*x*	[mm]	Equal to col. 22 until test SC2-350%; Equal to col. 78 from test SC2-400% till the end
96	South wall base shear | *V*_b,S_*(inertia force including the non-oscillatory mass)*	*x*	[kN]	Inertia force of the South wall plus half of the inertia forces of the East, West and interior walls
97	North wall base shear | *V*_b,N_*(inertia force including the non-oscillatory mass)*	*x*	[kN]	Inertia force of the North wall plus half of the inertia forces of the East, West and interior walls
98	Overall base shear | *V*_b,TOT_*(inertia force including the non-oscillatory mass)*	*x*	[kN]	The sum of the products of each accelerometer reading with the associated mass
99	Gable-roof assembly inertia force | *F*_R_	*x*	[kN]	The sum of the products of each accelerometer reading with the associated mass above the floor diaphragm (excluding the South chimney)
100	South wall base shear | *V*^0^_b,S_*(inertia force without the non-oscillatory mass)*	*x*	[kN]	Col. 96 minus the product of col. 60 times mass of 4106 kg
101	North wall base shear | *V*^0^_b,N_*(inertia force without the non-oscillatory mass)*	*x*	[kN]	Col. 97 minus the product of col. 59 times mass of 4282 kg
102	Overall base shear | *V*^0^_b,TOT_*(inertia force without the non-oscillatory mass)*	*x*	[kN]	Col. 98 minus the sum of the products of col. 60 and 59 with the masses 4106 and 4282 kg, respectively
103	Inertia force for top half part of the gable-roof assembly | *F*^0^_R_	*x*	[kN]	The sum of the products of each accelerometer reading with the associated mass above the gables mid-height

Finally, Table 8 lists the quantities found in the last two columns, No. 104 and 105. These are displacement and acceleration time histories retrieved from the video analysis of the South chimney at the fracture level above the roofline. Accelerations and displacements are provided in units of g and mm, respectively, while forces are expressed in units of kN.

**Table 8:** Displacement and acceleration time histories retrieved from the video analysis of the South chimney at the fracture level above the roofline (+3.78 m): matrix columns 104 and 105.

Col. No.	Measured quantity | location of the target point	Rec. DOF	UM	Tests
104	South chimney displ. at +3.78 m | Δ_m,C,S_ | *measured w.r.t. the shake table (target point found at mid-height of the free-standing part of the chimney, resulting after the fracture)*	*x*	[mm]	SC2-350%; SC2-400%; SC2-500%
105	South chimney accel. at +3.78 m | *a*_m,C,S_ | *measured in the longitudinal bldg. dir. (target point found at mid-height of the free-standing part of the chimney, resulting after the fracture)*	*x*	[g]	

The authors suggest that readers wishing to use the data to simulate the dynamic response of the building specimen should refer to column No. 82 for the input base accelerations (i.e., accelerations recorded at the foundation of the building). Accelerations recorded by sensors installed on the shake table were at a considerable distance from the foundation beam; hence, they might exhibit differences in amplitude. Such differences are not attributed to relative displacements (i.e., slide or uplift) of the building foundation concerning the shake table but to amplification caused by the presence of spurious rotational components that cannot be entirely excluded when controlling the tri-dimensional shake-table system under high-intensity input motions.

## Limitations

No significant limitations related to the data, except for the ones outlined in the sections dedicated to '*Missing instrument recordings*', '*Instrument saturation*', and '*Instrument malfunction*'.

## Ethics statement

The authors have read and followed the ethical requirements for publication in *Data in Brief* and confirm that the current work does not involve human subjects, animal experiments, or any data collected from social media platforms.

The reported work includes appropriate citations to all related publications that have influenced it. Furthermore, this article contains information not previously published in any other journal.

## CRediT authorship contribution statement

**Stylianos Kallioras:** Methodology, Software, Validation, Formal analysis, Investigation, Data curation, Writing – original draft, Visualization. **António A. Correia:** Methodology, Software, Formal analysis, Investigation, Resources, Data curation, Writing – original draft, Visualization, Supervision. **Paulo X. Candeias:** Methodology, Software, Formal analysis, Investigation, Data curation. **Alfredo Campos Costa:** Resources, Supervision. **Francesco Graziotti:** Conceptualization, Methodology, Resources, Writing – review & editing, Supervision, Project administration, Funding acquisition.

## Data Availability

Collapse shake-table testing of a full-scale clay-URM building with chimneys (LNEC-BUILD-3) (Original data) (www.betestdata.eu)
